# Investigating preferences for mosquito-control technologies in Mozambique with latent class analysis

**DOI:** 10.1186/1475-2875-10-200

**Published:** 2011-07-21

**Authors:** Rachel A Smith, Victoria C Barclay, Jill L Findeis

**Affiliations:** 1 Department of Communication Arts & Sciences and the Methodology Center, the Pennsylvania State University, University Park, PA, USA; 2 Center for Infectious Disease Dynamics, the Pennsylvania State University, University Park, PA, USA; 3 Department of Biology, the Pennsylvania State University, University Park, PA, USA; 4 Department of Agricultural Economics & Rural Sociology and Population Research Institute, the Pennsylvania State University, University Park, PA, USA

## Abstract

**Background:**

It is common practice to seek the opinions of future end-users during the development of innovations. Thus, the aim of this study is to investigate latent classes of users in Mozambique based on their preferences for mosquito-control technology attributes and covariates of these classes, as well as to explore which current technologies meet these preferences.

**Methods:**

Surveys were administered in five rural villages in Mozambique. The data were analysed with latent class analysis.

**Results:**

This study showed that users' preferences for malaria technologies varied, and people could be categorized into four latent classes based on shared preferences. The largest class, constituting almost half of the respondents, would not avoid a mosquito-control technology because of its cost, heat, odour, potential to make other health issues worse, ease of keeping clean, or inadequate mosquito control. The other three groups are characterized by the attributes which would make them avoid a technology; these groups are labelled as the bites class, by-products class, and multiple-concerns class. Statistically significant covariates included literacy, self-efficacy, willingness to try new technologies, and perceived seriousness of malaria for the household.

**Conclusions:**

To become widely diffused, best practices suggest that end-users should be included in product development to ensure that preferred attributes or traits are considered. This study demonstrates that end-user preferences can be very different and that one malaria control technology will not satisfy everyone.

## Background

Successful diffusion of mosquito-control technologies for malaria is complex, involving several technical, operational, economic, and social factors. One way to improve diffusion is to design technologies that are sympathetic to the socio-economic landscape. Indeed, it is common practice to seek the opinions of future end-users during the development of innovations, allowing developers to include end-users' preferred attributes in the final innovation [1], with the hope of improving diffusion. If malaria control efforts were to develop technologies with the greatest chance of uptake, two related questions arise: "What do users prefer?" and, "What attributes concern end-users enough to avoid technologies with them, and instead, opt for those without them?"

Potential end-users' preferences likely vary; Reported experiences of bed nets (treated or not) are one example of variance in these preferences. The benefits of reducing the nuisance associated with mosquitoes has been preferred by some [2] and dismissed by others who say that mosquitoes still bite them before bed time [3]. Several reasons have been given for failing to sleep under a bed net: discomfort, particularly the heat, of sleeping under one [4]; limited access to nets [5]; and cost of purchasing nets [6,7]. Attributes of technologies, such as the texture, mesh size, shape, and colour of insecticide-treated bed nets (ITNs), have also affected bed net use and acceptability [8-10]. Nets in colours that show dirt easily, such as white, have been avoided [10]. In addition, net materials that wrinkle and shorten with use are also avoided, because these changes in the material make it more difficult to keep them tucked in place so that mosquitoes cannot get in [8]. In response to the concerns about discomfort, particularly heat in hot climates, bed net manufacturers have tried to improve ventilation with larger-sized mesh [8]. Interestingly, users are wary about nets with larger mesh, because they may allow mosquitoes inside [8]. When considering ITNs as a long-term control strategy, people may need to acquire multiple nets in a lifetime. Thus, it is easy to fix issues such as colour and size [9], and doing so may assist with initial uptake [10] and long-term markets [9].

People's preferences for malaria technologies also have ramifications for the deployment of existing technologies and development of new technologies. For example, late life acting (LLA) insecticides [11], which focus on killing older, infected mosquitoes, have the potential to slow insecticide resistance and to eradicate malaria, but at the perceived cost of nuisance bites from young mosquitoes who cannot infect humans with malaria. Researchers also still need to determine which potential end-users are concerned about cost (and different aspects of cost), and which of the cost-concerned end-users are also concerned about other attributes, such as bad odours and washability.

Indeed, it is likely that users can be clustered into different groups based on common characteristics, such as their preferences for an innovation's attributes [1]. If this is the case with malaria technologies, then such profiles would provide a means by which to consider the costs and benefits of designing technologies to meet varying preferences. Some preferences may take more time and money, thus delaying its appearance and increasing its price; it may not make sense to incorporate these preferred attributes if only a small segment of the population prefers them. Other preferences may already be addressed with existing technologies, individually or in combination. A recent call to combine existing interventions together to reduce malaria more quickly and completely [5] has a better chance of success if there is a thorough understanding of users' preferences.

It is possible that some covariates may predict people's membership in different latent classes. Existing research shows that people living in areas of high populations of year-round mosquitoes report using bed nets regardless of whether the nets have the traits they prefer [3,8], but for those with low or seasonal exposure, unfavourable attributes of nets were a stronger reason for a lack of use [8]. People with higher education or economic means may have more preferences in malaria technologies, either because they are more aware of these options or because they can be selective. These covariates are tested for their ability to predict membership in different attribute-preference groups.

Thus, the aim of this study is to investigate latent classes of end users based on their preferences in malaria technologies attributes. Further, it explores which current technologies meet these preferences, and the consequences of adapting current or developing new technologies to meet them.

## Methods

### Study site

Surveys were conducted in five rural villages in Mozambique (Lione, Lucucho, Nhamane, Ntapo, and Somba). The sampling area for each village was limited to households within a 4 km^2 ^area, centered at the heart of the community (as confirmed by the local leadership). Most villagers are subsistence farmers. Malaria is transmitted year-round in Mozambique, with a seasonal peak during the rainy season, from October to April depending on location. Mozambique was chosen because it is a representative country with a high malaria burden.

### Data collection and sampling

Participants (*N *= 271, 54% female) were recruited from households that were randomly selected from geographic maps of each village, using individual dwellings/compounds as the indicators of households. Interviews were conducted over multiple days during the dry season. Everyone invited into the study participated.

Interviewers from the region who were fluent in Portuguese and local languages helped to guide respondents through the survey (91% of respondents had limited understanding of Portuguese). Photographs were used to illustrate mosquito-control technologies. The survey included information on their reported use of mosquito-control technology in the past week (any use of bed nets, sprays, aerosols, or screens), the attributes that would lead them to avoid one technology and use another instead, perceived efficacy to use mosquito-control technologies and to prevent malaria, malaria beliefs and experiences, and socio-demographic variables. All interviewers received training before and during the data collection. The data were checked by a supervisor each night. Interviewers (one male and one female) approached each household and asked to talk to the male and female heads of the household as identified by the household members (some households only had females in this position). Participants classified themselves as Roman Catholic (35%), Muslim (33%), Protestant (16%), practicing other religions (15%), or reported no religious affiliation 1%). On average, they were 39 years old (*SD *= 16.48).

### Ethical considerations

Each interviewer took the interviewee of the same gender to a private place and obtained consent from the respondents before starting the survey. Interviewers guided respondents through the survey, to gain information from those with low reading abilities. The institutional review board of Pennsylvania State University approved the protocols for this project.

### Analysis of data

The data analysis was conducted with SAS 9.2. Latent class analysis was completed with PROC LCA 1.2.5 beta [12]. Latent class analysis (LCA [13]) is a statistical model used to identify underlying mutually exclusive and exhaustive subgroups of individuals with shared characteristics. LCA is used in this study to empirically test whether meaningful latent classes of people can be identified based on their preferred attributes for technologies. LCA tests whether individual characteristics predict the odds of membership in one class relative to a reference class. These characteristics are included in the model as covariates, and the uncertainty associated with individuals' latent class membership is taken into account. In other words, a latent class measurement model and a logistic regression model predicting classes from covariates are modelled simultaneously without actually assigning individuals to a particular latent class [12].

## Results

### Technology preferences

Participants' preferences in mosquito-control technologies are shown in Table 1. The most frequently listed concern that would make participants avoid using one malaria control product over another is inadequate mosquito bite control (*n *= 108, 39.9%). Due to the infrequency of responses expressing a concern for genetic modification (*n *= 13, 4.8%), this choice was eliminated from the latent class analysis. (Responses could reflect lack of concern for genetic modification or lack of knowledge of genetic modification.)

**Table 1:** Descriptive statistics of preferences in mosquito-control technologies used as indicators of latent classes (n = 271; 54.2% female)

Would this make you avoid using one malaria control product over another?	Yes, n (%)
Mosquitoes still bite me.	108 (39.9)
It is too expensive.	94 (34.7)
It makes it hotter when I'm sleeping.	88 (32.5)
It smells worse.	83 (30.6)
It cannot be washed.	68 (25.1)
Mosquitoes still make noise.	67 (24.7)
It causes other illnesses or makes other health conditions worse.	45 (16.6)
It gets dirty easily.	37 (13.7)

### Preference classes

Models with two to six latent classes (using 100 sets of random starting values for each) are tested. As seen in Table 2, a four-class model best fits the data. The item-response probabilities appear in Table 3, and are used to interpret these classes.

**Table 2:** Comparison of latent class models

Number of classes	***G***^**2**^	*df*	AIC	BIC	**Entropy R**^**2**^
2	417.19	492	455.19	523.63	0.83
3	339.48	482	397.48	521.95	0.84
**4**	**288.00**	**472**	**366.00**	**506.48**	**0.85**
5	265.41	462	363.41	539.91	0.87
6	243.60	452	361.60	574.13	0.91

*Note*. Boldface type indicates the selected model, which has the lowest AIC and BIC. df = degrees of freedom; AIC = Akaike's information criterion; BIC = Bayesian information criterion.

**Table 3:** Probability of reporting each characteristic given latent class membership

	No preferences (48%)	Bites (34%)	By-products (16%)	Multiple concerns (10%)
Mosquito bites	0.01	**0.70**	0.53	**0.89**
Expensive	0.01	0.57	0.39	**1.00**
Heat	0.14	0.23	**0.77**	**0.70**
Odour	0.03	0.37	**0.75**	**0.91**
Unwashable	0.14	0.28	0.17	**0.81**
Mosquito noise	0.00	0.38	0.24	**0.91**
Side effects	0.00	0.00	**0.61**	**0.70**
Dirties easily	0.01	0.09	0.21	**0.66**
Female	0.47	0.50	1.00	0.19

Participants in the smallest class (10%), labelled *multiple concerns*, are likely to avoid using one malaria product over another for the following reasons: they would still be bitten by mosquitoes, they would still hear the mosquitoes making noise, it was expensive, it made the person hotter when sleeping, it had an odour, it was not washable, and it made other illnesses worse (i.e., caused side effects). It is also more likely that men, versus women, are in the multiple-concerns class. Participants in the next biggest class, *by-products *(16%), are likely to be females and are likely to avoid a malaria product based on heat, odour, and consequences for other illnesses (i.e., side effects). Participants in the *bites *class (34%), as the label suggests, are likely to avoid a product if they would still be bitten by mosquitoes. The largest class, no preferences (48%), are not likely to avoid one malaria product over another because of these attributes. These last two classes--bites and no preferences--were not distinguishable by gender.

### Covariates of group membership

Multiple covariates were investigated: the number of days respondents were sick with malaria in the past year, perceived seriousness of malaria for the household, the number of months they faced financial stress in the past year, perceived self-efficacy to engage in malaria prevention, likelihood of trying new mosquito-control technologies (these variables were standardized), reported use of mosquito-control technology (bed nets, sprays, aerosols, or screens) in the past week, and if they were able to read and write (1 = *yes*, 0 = *no *for these last two variables). The results are presented in Table 4.

**Table 4:** Covariate analysis with no-preference members as the comparison class

	Bites	By products	Multiple concerns	Change in LL^2^
	**OR (β)**	**OR (β)**	**OR (β)**	

Malaria sick days	1.54 (0.42)	1.76 (0.57)	1.58 (0.46)	4.42
Seriousness household	1.50 (0.41)	0.80 (-0.22)	2.51 (0.92)	13.37*
Able to read and write	0.43 (-0.82)	0.17 (-1.80)	1.76 (0.56)	17.95*
Financial stress	0.97 (-0.03)	0.87 (-0.14)	0.64 (-0.44)	1.87
Prevention in past week	2.14 (0.76)	1.87 (0.63)	1.16 (0.15)	4.37
Self efficacy	0.61 (-0.49)	0.91 (-0.09)	1.94 (0.66)	8.95*
Try new controls	1.65 (0.50)	1.19 (0.17)	1.93 (0.66)	8.94*

* *p *< .05.

Four covariates differed between classes: perceived seriousness, literacy, self-efficacy, and trying new mosquito-control technologies. In comparison to the no-preferences class, members of the bites and multiple-concerns classes perceived malaria as a more serious concern for their households, while members of the by-products class perceived malaria as less of a household concern. Compared to the no-preferences class, members of the bites and by-product classes had lower odds of being able to read and write and perceiving themselves as able to use mosquito-control technologies, but the multiple-concerns class had higher odds of literacy and self-efficacy.

Three classes, bites, by-products, and multiple concerns, had increased odds of showing interest in trying new mosquito-control technologies. Sick days, financial stress, and reported use of a bed net, spray, aerosol or screen in the past week did not vary between classes.

## Discussion

This study showed that users' preferences in mosquito-control technologies varied, and people could be categorized into latent classes based on technology preferences. The analysis showed that four latent classes could be identified based on shared preferences in mosquito-control technologies. The largest class, constituting almost half of the respondents, would not avoid a mosquito-control technology because of its cost, heat, odour, potential to make other health issues worse, ease of keeping clean, or mosquito control. The other three groups could be characterized by the attributes which would turn them off from a technology, labelled as the bites class, by-products class, and multiple-concerns class.

Of note, only two of these classes - bites and multiple concerns - had higher probabilities of avoiding technologies that lacked mosquito bite control. Of technologies available, aerosols, screens, personal repellents, indoor residual sprays (IRS; e.g., pyrethroids, carbamates, organophosphates, and organochlorines), and bed nets provide a means by which to avoid mosquito bites. If LLA-insecticides, which only kill old infectious mosquitoes [14], were promoted to these classes it may be beneficial to combine those efforts with a secondary means (e.g., bed nets) by which to avoid nuisance bites.

Members of the by-products class are likely to avoid technologies that make other health conditions worse, smell badly, and make sleeping hotter. One could imagine LLA-insecticides being deployed in households belonging to this class, as they would be less concerned with being bitten, and more concerned that that the technology did not irritate other health conditions, have a bad smell or require sleeping under a net to be effective. Perhaps households belonging to this class would prefer an insecticide that killed mosquitoes in ways other than with chemicals. For example, fungal bio-pesticides are already known to generate the required phenotypes of an LLA insecticide [11,15]. These insecticides are based on oil-formulated spores of entomopathogenic fungi applied to surfaces on which adult mosquitoes rest after blood feeding [16]. Fungal bio-pesticides are environmentally friendly, not known to be irritants and do not have an odour. In fact, it may be possible to add a pleasant smell to products to improve uptake.

The by-products class may also be best targeted by chemotherapeutic interventions, in which the goal is not to control mosquitoes or to prevent bites, but to prevent clinical disease. Members of this class may be more willing to be immunized with a new vaccine [17,18] or be treated with anti-transmission drugs [19,20], assuming that they believe that the vaccine and drugs do not have side effects.

The smallest class was the multiple-concerns class. Members of this class are likely to avoid technologies if they are costly, hot, malodorous, easy to dirty, unwashable, and unable to control mosquito bites and noise. Members of this class are also likely to be males. The most appropriate technology for this class is likely to be a potent, inexpensive, odourless insecticide sprayed on the walls of a house (as opposed to nets). Unfortunately, insecticides that are the most potent to mosquitoes are also the most potent selectors for mosquito resistance [21].

Since the multiple concern class was so small, one could wonder if it is worth the time and expense of trying to develop a technology to meet these preferences. Importantly, the multiple-concerns class were those people with a higher likelihood of literacy and efficacy. It is possible that members of this class may be targeted with educational and behavioural change campaigns. Indeed, one may argue that three of the attributes - washability, heat, and side-effects, may be misunderstandings about technologies. The relative efficacy and sustainability of addressing users' preferences in design or education is an important question worth future investigation.

The findings support the idea of developing and maintaining a portfolio of mosquito-control technologies instead of creating and distributing only one across the country. This diversification strategy is used in other technology markets (e.g., mobile phones with a variety of technologies and price points). The ramifications of maintaining diversity in public health technologies is to consider audience preferences, clusters of preferences, and how they vary by geographic location more thoroughly throughout the intervention process. Of note, the findings from this study are closely aligned with those found in previous studies (e.g., 39% reported heat discomfort [5] as compared to 32.5% of participants in this study). The benefit of profiles and audience segmentation is that it allows for identifying (a) clusters of preferences, (b) size of profile classes, and (c) covariates of class membership. With this level of precision, more nets may be sent to some areas and more sprays to another based on users' preferences. Assuming equivalency in public health protection, this approach and framework allows for more precisely aligned audience segmentation and distribution. Additionally, if the preferred technology is not available for a particular area, understanding the profiles of preferences facilitates the development of user-relevant messages for education and persuasion campaigns.

### Covariates of group membership

It is important to note that the number of sick days a person experienced last year due to malaria, their use of mosquito-control technologies (bed nets, IRS, aerosols, or screens), and financial stress were not significant covariates of membership in the preference classes. This may be, in part, because everyone in the community felt some financial stress, and money may be a critical barrier to the uptake of mosquito-control technologies [10].

Literate, efficacious, innovative, concerned participants had higher odds of membership in the multiple-concerns class, relative to the no-preferences class. These two classes are the most extreme in the preference profiles, from everything to nothing. It is interesting that those with the characteristics most strongly associated with adoption of new behaviours [22], are those most likely to find a number of attributes concerning enough to avoid one technology and use an alternative. This underscores why it is important to know about such preferences and potentially incorporate them into new versions of mosquito-control technologies.

In addition to technology preferences, future research should attend to users' preferences in cost and delivery methods, because meeting these preferences can affect uptake and distribution [23,24]. Access in general is a challenge in Mozambique. In a recent attempt to distribute bed nets with vouchers in Mozambique, only 68% of eligible households received a voucher - after including new districts because some were inaccessible due to flooding [25]. Of those receiving a voucher, almost all (90%) redeemed it.

### Limitations

The findings from this study have limitations. First, participants were asked which attributes would lead them to avoid one mosquito-control technology and to select another instead. The technology, itself, was not specified, but left in the abstract. Although the answers were likely primed by the previous question on their use of existing technologies in the past week, some specificity is needed before design considerations may be finalized. Cost is a relevant example: the financial amount associated with 'expensive' may vary among participants. This type of analysis provides a useful, initial screen for issues to investigate, which can be refined later with exact specifications. Second, participants' current use of mosquito-control technologies was not high, which can be explained by the fact that the surveys were completed during the dry season. None of the participants indicated a lack of familiarity with existing technologies, but it is possible that such answers are biased by social desirability. Third, the adoption of new technologies and their diffusion through social systems is a complex phenomenon of which user preferences are but one, albeit important, consideration (Rogers, 2003).

## Conclusions

To become widely diffused, best practices suggest that end-users should be included in product development to ensure that preferred attributes or traits are considered [22]. Historically, it was assumed that for malaria eradication, one approach could succeed everywhere, as long as it was taken with "military precision" [26]. This study demonstrates that end-user preference can be very different and one technology will not fit all in malaria control. In reality, it is likely to be difficult to collect end-user profiles country-wide. However, one of the biggest concerns currently facing the 32 countries eliminating malaria is that elimination will not be possible if transmission is not prevented in the most isolated and marginalized communities [27]. By definition, an elimination programme must go the last mile and successfully implement culturally sensitive technologies, and considering end-user profiles in those communities may be particularly useful [3].

## Competing interests

The authors declare that they have no competing interests.

## Authors' contributions

RS and JF conceived of the study, and participated in its design and coordination. RS performed the statistical analysis. RS and VB drafted the manuscript. All authors read and approved the final manuscript.
